# Dealing with Feelings: Characterization of Trait Alexithymia on Emotion Regulation Strategies and Cognitive-Emotional Processing

**DOI:** 10.1371/journal.pone.0005751

**Published:** 2009-06-03

**Authors:** Marte Swart, Rudie Kortekaas, André Aleman

**Affiliations:** NeuroImaging Center, University Medical Center Groningen, University of Groningen, Groningen, The Netherlands; James Cook University, Australia

## Abstract

**Background:**

Alexithymia, or “no words for feelings”, is a personality trait which is associated with difficulties in emotion recognition and regulation. It is unknown whether this deficit is due primarily to regulation, perception, or mentalizing of emotions. In order to shed light on the core deficit, we tested our subjects on a wide range of emotional tasks. We expected the high alexithymics to underperform on all tasks.

**Method:**

Two groups of healthy individuals, high and low scoring on the cognitive component of the Bermond-Vorst Alexithymia Questionnaire, completed questionnaires of emotion regulation and performed several emotion processing tasks including a micro expression recognition task, recognition of emotional prosody and semantics in spoken sentences, an emotional and identity learning task and a conflicting beliefs and emotions task (emotional mentalizing).

**Results:**

The two groups differed on the Emotion Regulation Questionnaire, Berkeley Expressivity Questionnaire and Empathy Quotient. Specifically, the Emotion Regulation Quotient showed that alexithymic individuals used more suppressive and less reappraisal strategies. On the behavioral tasks, as expected, alexithymics performed worse on recognition of micro expressions and emotional mentalizing. Surprisingly, groups did not differ on tasks of emotional semantics and prosody and associative emotional-learning.

**Conclusion:**

Individuals scoring high on the cognitive component of alexithymia are more prone to suppressive emotion regulation strategies rather than reappraisal strategies. Regarding emotional information processing, alexithymia is associated with reduced performance on measures of early processing as well as higher order mentalizing. However, difficulties in the processing of emotional language were not a core deficit in our alexithymic group.

## Introduction

Alexithymia, or “no words for feelings”, is a personality trait characterized by difficulties in emotion regulation, difficulties in identifying, describing and communicating feelings, difficulties in differentiating feelings from bodily sensations and diminished affect-related fantasy [1], [2]. Alexithymia has been reported to be a risk factor for a variety of medical and psychiatric disorders like substance use disorders, somatization, anxiety and depression [3], and even schizophrenia [4]. Moreover, alexithymia reduces life satisfaction [5]. In a large sample in the general Finnish population, the prevalence rate of alexithymia was around 10% [6]. Unraveling the psychological mechanisms underlying alexithymia may have important clinical and societal implications.

Even though several studies have investigated the underlying mechanisms of emotional processing in alexithymia, using a variety of tasks [e.g. 7]–[11; for an overview see 12]; the results remain equivocal. On a basic emotional-perceptual level of processing, Suslow [10] found that the ‘difficulty describing feelings’ score on the 20-item Toronto Alexithymia Scale (TAS-20) [13] was correlated to a facilitation effect in a priming paradigm for negative words, consistent with an enhanced automatic processing of affective information. Contradictory to these results, Vermeulen et al. [11] showed that individuals with high scores on alexithymia are less prone to process emotional information at an automatic level, as was investigated in a different priming paradigm, in which negative and positive primes (resp. angry and happy faces) were included. Only the presentation of the angry face resulted in a lower priming effect for participants with high alexithymia scores [11].

Lane and colleagues [7], [8], however, reported that high alexithymic individuals perform worse on recognizing all basic emotions in an emotional perception task. This task consisted of four subtasks in which pairs of sentences and words, faces and words, sentences and faces and faces and photographs of scenes had to be matched for emotion. In line with these findings one would expect to see a lower priming effect of both happy and angry faces in the priming paradigm from Vermeulen et al. [11]. In summary, emotional processing on a basic perceptual level seems to be altered in alexithymia but the results are inconsistent.

On a cognitive-emotional level in which cognition plays a more prominent role, such as memory for emotions or learning associations between emotions and words, Luminet et al. [9] compared people with high versus low alexithymia levels on a memory task in which participants had to recall emotional and neutral words. They had to indicate if they only ‘knew’ that they had seen the word in the list, i.e. without retrieval of any details or that they ‘remembered’ the word with details, for example, the position of the word in the list. Alexithymics gave less ‘remember’ responses for emotional words but responded the same for neutral words. Groups did not differ in ‘know’ responses for emotional and neutral words. Thus, it appears that cognitive-emotional processing differs between individuals with high and low alexithymia scores.

Mentalizing, a cognitive skill also known as theory of mind (TOM), refers to understanding that others have beliefs, desires and intentions different from the self [14]. Mentalizing is impaired in psychiatric disorders which are associated with alexithymia like schizophrenia [4], [15] and Asperger's syndrome [16]. Healthy people with high alexithymia scores are also impaired in mentalizing and show reduced brain activation of medial frontal areas during mentalizing [17]. The latter study examined the participants' ability to infer what other people think but not what they feel. This latter ability, *emotional* mentalizing, has not been studied but we expect it to be impaired in alexithymia.

Each of the studies mentioned above focused on one stage of information processing in alexithymic individuals: basic-emotional, cognitive-emotional and mentalizing. It seems that high and low alexithymics differ on all three stages of emotional information processing but results are ambiguous. This ambiguity could be due to the participation of subjects from different populations and the use of different inclusion criteria in the aforementioned studies.

The main purpose of the present study was to investigate differences in stages of emotional processing in individuals with high versus low verbalizing scores on the Bermond-Vorst Alexithymia Questionnaire (BVAQ) [18]. Both auditory-verbal and visual modalities at different levels of emotional processing were investigated. We included measures of early perception of facial emotional expressions, and recognition of emotional prosody. In addition, we included tasks with stronger cognitive demands. Aleman [19] proposed that learning to verbalize emotions requires development of an association between particular affective states and particular words and that alexithymics are less able to make these associations. Therefore, we included a task in which subjects learned associations between words and emotional facial expressions. Additionally, to investigate whether alexithymia is a deficit of emotional awareness in general (independent of modality) or whether it is more pronounced for language-related processes, we included tasks with emotional linguistic stimuli. Moreover, because deficits in thinking about and interpreting emotions is central to alexithymia, we included an emotional mentalizing task to probe the meta-cognitive level. To the best of our knowledge, no studies have been reported yet on emotional mentalizing abilities in alexithymics. We expected an inverse relationship between alexithymia and emotional mentalizing capacity.

In addition, to shed light on behavioral emotional processing, we aimed to clarify differences in emotion regulation strategies, as described by Gross & John [20]. To this end, we included questionnaires measuring reappraisal, suppression and expressivity. We were primarily interested in the aspect of “no words for feelings” to examine if alexithymia is related to language-related processes. Therefore, we initially selected participants based on extreme scores on the verbalizing subscale of an alexithymia questionnaire, which specifically assesses the difficulty in verbalizing one's feelings. We anticipated that subjects with high scores on the verbalizing scale would also have higher scores on the other scales of the alexithymia questionnaire.

## Methods

### Participants

A total of 493 university students filled in the verbalizing scale of the Bermond Vorst Alexithymia Questionnaire (BVAQ) [18]. Nineteen individuals with score≤17 and 24 with score≥26 and who gave permission to be contacted for further research, were initially selected for this study. These cutoff values were chosen to generate subgroups roughly corresponding to the lowest and highest quartiles. Participants filled in the complete BVAQ when they came for the experiment. At this time, the participants from the low alexithymia group were excluded if they scored above the overall mean verbalizing score of the 493 students (score>20.97). To ensure that the high alexithymia group was robust, reliable and reproducible, we only included participants in the high group if they still scored≥26. Additionally, the mean of the high group had to be more than 1.5 SD (score>29.73) above the overall mean of the verbalizing scale. After this second selection, we included eighteen participants in the low alexithymia group (eleven females, mean age 19.3 years, SD 1.0) and sixteen in the high (nine females, mean age 20.1 years, SD 1.7). Due to the more stringent inclusion criteria for the high group, more individuals had to be excluded from this group.

Groups differed significantly on the second measurement of the verbalizing scale (F(1,32) = 174.89, p<0.001).

The study was approved by the Ethics Committee Psychology of the University of Groningen. All participants gave their written informed consent. Participants were paid € 12 for participation.

### Questionnaires

#### Bermond-Vorst Alexithymia Questionnaire

The BVAQ is a 40-item self-report scale, which is subdivided into 5 scales (8 items per scale), comprising the alexithymia features as defined by Nemiah and Sifneos [21] and Sifneos [1], namely verbalizing, analyzing, identifying, emotionalizing and fantasizing. Previous studies have shown that the BVAQ has good psychometric properties and that the 5-factor structure of the BVAQ is supported by factor-analyses [18], [22]–[24]. Answers are scored on a 5-point scale (1 = certainly does not apply to me, up to 5 = certainly applies to me). Higher scores indicate more alexithymic. Participants were selected on the verbalizing scale of the Bermond-Vorst Alexithymia Questionnaire (BVAQ) [18]. An example of the verbalizing scale is “I find it difficult to verbally express my feelings”. At the time of testing, they were also asked to fill in the complete BVAQ. Bermond and colleagues have made a second order distinction, in which they distinguish a cognitive component, which comprises the verbalizing, analyzing and identifying subscales, and an affective component, consisting of the emotionalizing and fantasizing subscales. This two-factor structure has been validated in six languages and seven populations [25]. The correlation between the cognitive component of the BVAQ and the Toronto Alexithymia Scale (TAS-20) [13], which also targets the cognitive component of alexithymia, is high (r = 0.80) [18].

#### Emotion Regulation Questionnaire

Emotion regulation was measured with the Emotion Regulation Questionnaire (ERQ) [20]. This scale measures two emotion regulation strategies: cognitive reappraisal and expressive suppression. Cognitive reappraisal is a cognitive strategy involving reinterpretation of a potentially emotion-eliciting situation into a situation with a different emotional impact [26]. Expressive suppression is a way of response modulation involving inhibition of emotion-expressive behavior [27]. Examples of this questionnaire are “I control my emotions by changing the way I think about the situation I'm in” (reappraisal), “I control my emotions by not expressing them” (suppression). The scale consists of 10 items (6 reappraisal items, 4 suppression items). Lower reappraisal and higher suppression scores indicate more problems with emotion regulation.

#### Berkeley Expressivity Questionnaire

Emotional expressivity was measured with the Berkeley Expressivity Questionnaire (BEQ) . This questionnaire assesses three facets of emotional expressivity: negative expressivity (NE) (6 items), positive expressivity (PE) (4 items), and impulse strength (IS) (6 items). The questionnaire measures the degree to which both positive and negative emotions are expressed behaviorally and also the general strength of the emotional impulses. Examples of items are “It is difficult for me to hide my fear” (NE), “When I'm happy, my feelings show” (PE), “My body reacts very strongly to emotional situations” (IS) [28]. Items can be rated on a scale ranging from 1 (strongly disagree) to 7 (strongly agree). Higher scores indicate higher degrees to which emotion response tendencies are expressed as manifest behavior and a higher general strength of these tendencies.

#### Positive and Negative Affect Schedule

Positive and negative affect were measured with the Positive Affect Negative Affect Schedule (PANAS). The PANAS measures the current affective state. Positive affect (PA) reflects the extent to which a person feels enthusiastic, active and alert (examples: “interested” and “excited”). Negative Affect (NA) is a general dimension of distress, two items of this subscale are “nervous” and “upset” [29]. This scale consists of 10 positive affect items and 10 negative affect items. Higher scores indicate stronger affect (either positive or negative).

#### Empathy Quotient

Empathy was measured with the Empathy Quotient (EQ) [30]. This questionnaire measures the ability to what extent one is able to tune into how someone else is feeling, or what someone else might be thinking. An example of an item is “I am good at predicting how someone will feel.” This scale comprises 60 items, including 20 filler items. A high score means a high degree of empathy.

### Tasks

#### Micro Expression Training Tool

In the Micro Expression Training Tool (METT) [31] participants had to learn to recognize micro expressions in faces. Micro-expressions are very brief (15 ms) facial expressions, beginning and ending with a neutral expression. In this task seven different emotions were shown: sadness, anger, surprise, fear, disgust, contempt and happiness. The participant was first trained and then allowed to practice to learn to recognize micro-expressions of emotions. The training consisted of four pairs of faces expressing commonly confused emotions (anger/disgust, contempt/happy, fear/surprise, fear/sadness). Each trial started with two neutral faces, which simultaneously transformed in slow motion (4 s) into two commonly confused emotions. In each trial, the faces transformed four times from neutral to the emotional expressions and back to neutral, ending in an emotional expression. The spoken information about the expressions from the Ekman software [31] was not presented. In the practice session (42 trials), a micro-expression was presented and the participant had to select the corresponding emotion out of seven emotions. Feedback was given after each response. Finally, a test of micro-expression recognition was performed consisting of 14 trials (two trials per emotion), similar to the practice session but without feedback. Accuracy scores of the recognition test were used for statistical analysis. Due to technical problems, data were not available for one subject.

#### Associative Learning Task

Two associative learning tasks were administered (using E-prime software [32]). The tasks were based on the tasks described by Exner et al. [33]. Both tasks consisted of six pictures of basic facial emotional expressions from the Ekman and Friesen series [34] and six words. One of the tasks concerned identity and the other emotion. In the identity learning trials, six words (hat, scissors, carpet, basket, table, stone) paired with neutral expressions from different people (three male and three female faces) were shown for a maximum of 30 seconds each. Participants were instructed to memorize each pair. In the recall trial, that immediately followed the learning trial, participants had to match the correct face out of the six faces to each word subsequently. This sequence was presented six times in random order amounting to 36 trials. After the participant recalled all six pairs correctly or after six learning and recall trials, the task was terminated. The emotional learning task was the same as the identity learning task except for the pictures of the faces and the words (car, newspaper, paper, bag, football, and chair). In this task, the face showed the six basic emotional expressions (anger, fear, disgust, sadness, happiness and surprise) of one woman. In the recall trial, the participant had to match the correct emotional expression to each word. The number of correct pairs was used for statistical analysis.

#### Affective Prosody Task

Participants were asked to identify the emotion expressed through the prosody or semantics of a spoken sentence. This task consisted of sentences with an emotional content (happy, sad, angry, anxious) pronounced with an incongruent emotional tone of voice (happy, sad, angry, anxious). The sentences were pronounced by two professional actors, a male and a female voice, to control for individual and/or gender differences in affective prosody [35]. The sentences were of approximately equal length and were presented via two speakers by a computer at a rate of one sentence per 20 seconds. During listening, the emotion labels from which the participant could choose (fear, anger, happiness and sadness) were presented on the computer screen. In the prosody condition, participants had to attend to the affective tone of voice and ignore the incongruent affective semantic content. In the semantics condition, participants had to attend to the affective semantic content and ignore the affective tone of voice. Participants were instructed to make a response as soon as they identified the emotion expressed in the sentence, either based on content or tone of voice (‘prosody’) [35]. Accuracy and reaction times were used for statistical analysis. Due to a lack of correct responses in one condition, four participants were not included in this analysis.

#### Conflicting Beliefs and Emotions

We employed a Dutch translation of the task designed by Shaw and colleagues [36] to measure cognitive and emotional aspects of ‘theory of mind’. It consisted of eight vignettes, each concerning a short story involving a social situation of either exclusion or threat. Each story features two actors, A and B; A holds a true first order belief and B holds a false second order belief. Each belief is associated with an emotional state, one with positive and the other with negative valence. Each story was followed by six questions aimed at testing participants' understanding of the conflicting beliefs and the associated emotional states. Two first-order and two second-order questions were included. The first-order questions tested participants' ability to deduce from the story the belief and emotional state of actor A. The second order questions tested participants' understanding of the false belief of actor B on the thoughts of actor A as well as the by actor B perceived associated emotional state of actor A. Two control questions were included to test recall of the story and the making of inferences. Two blind raters scored the task independently and assigned 0 for a wrong response, 1 for a partially correct response and 2 for a correct response.

### Statistical Analyses

Statistical analyses were performed using Statistical Packages for the Social Sciences 14.0 [37]. All analyses were performed two sided. The alexithymic and non-alexithymic groups were compared on their verbalizing score with an independent t-test. Data from the BVAQ, ERQ, BEQ and EQ were analyzed with a multivariate analysis of variance (MANOVA) with the subscales of the questionnaires as dependent variables and Group as independent variable. The effect of alexithymia on the positive and negative affect scales were analyzed with an analysis of variance (ANOVA). Correlations between subscales of all questionnaires were analyzed by Spearman's rank correlation coefficients.

Differences between groups on reaction times (only correct trials) and accuracy scores of the associative learning task (identity and emotional learning) were both analyzed with analysis of variance (ANOVA).

Task performance on the semantic subtask and prosody subtask of the affective prosody task were analyzed with two MANOVAs with reaction times per emotion as dependent variables. Accuracy scores of this task were analyzed in the same way.

Accuracy scores on the METT task were compared with ANOVA.

The Conflicting Beliefs and Emotions vignettes were tested with a Kruskal-Wallis test with the separate mean scores on the vignettes as dependent variables.

## Results

### Questionnaires

MANOVA revealed a significant group effect on BVAQ, ERQ, BEQ and EQ scores (F(12,21) = 13.24, p<0.001). See Table 1 for descriptive statistics and group effects.

**Table 1:** Mean scores (S.D.) on questionnaires for the alexithymic and non-alexithymic groups.

	Alexithymic	Non-Alexithymic	F	P
**Bermond-Vorst Alexithymia Questionnaire**				
Verbalizing	32.44 (4.46)	13.83 (3.75)	174.89	0.001**
Analyzing	20.88 (5.80)	13.78 (4.54)	15.97	0.001**
Identifying	18.75 (4.88)	14.44 (4.00)	7.98	0.008*
Emotionalizing	21.94 (6.39)	19.67 (5.74)	1.19	0.28
Fantasizing	23.13 (5.40)	20.50 (7.96)	1.23	0.28
**Emotion Regulation Questionnaire (ERQ)**				
Reappraisal	25.63 (5.12)	30.22 (5.11)	6.85	0.01*
Suppression	17.06 (3.00)	10.39 (3.65)	33.42	0.001**
**Berkeley Expressivity Questionnaire (BEQ)**				
Total Positive Expressivity	17.69 (2.41)	22.83 (3.49)	24.42	0.001**
Total Negative Expressivity	18.69 (3.28)	25.33 (6.27)	14.43	0.001**
Total Impulse strength	21.31 (5.51)	27.56 (7.45)	7.56	0.01*
**Emotion Quotient (EQ)**				
Empathy scale	34.19 (14.47)	45.67 (10.13)	7.31	0.01*
Control items	11.31 (2.52)	12.39 (3.99)	0.86	0.36
**Positive and Negative Affective Scale (PANAS)**				
positive affect	29.63 (5.21)	30.44 (6.22)	0.17	0.68
negative affect	13.30 (3.79)	11.78 (2.34)	2.60	0.12

* significant at p<0.01 level (two-sided).

** significant at p<0.001 level (two-sided).

#### Alexithymia Questionnaire (BVAQ)

The alexithymic group not only scored higher on verbalizing (F(1,32) = 174.89, p<0.001), but also on identifying (F(1,32) = 7.98, p = 0.008) and analyzing (F(1,32) = 15.97, p<0.001). However, there were no group differences on the emotionalizing (p = 0.28) and fantasizing (p = 0.28) scales. We have to mention that the alexithymic group scored high on the cognitive component of the questionnaire but not on the emotionalizing component. Notably, the cognitive component of the BVAQ correlates highly with the TAS-20. This implies comparability between our sample and samples selected using the TAS-20 questionnaire.

The verbalizing subscale of the BVAQ was positively correlated with the ERQ subscales reappraisal (r = 0.46, p = 0.006) and suppression (r = 0.82, p<0.001), and negatively correlated with the BEQ positive expressivity (r = −0.73, p<0.001), BEQ negative expressivity (r = −0.67, p<0.001), BEQ impulse strength (r = −0.46, p = 0.007) and EQ empathy scale (p = 0.04).

#### Emotion Regulation Questionnaire

The high alexithymic group scored lower on the reappraisal (F(1,32) = 6.85 p = 0.013) and higher on the suppression scale of the ERQ (F(1,32) = 33.42, p<0.001).

#### Berkeley Expressivity Questionnaire

The high alexithymic group had lower ratings on the positive (F(1,32) = 24.42, p<0.001) and negative (F(1,32) = 14.43, p = 0.001) expressivity and impulse strength (F(1,32) = 7.56, p = 0.01) dimensions of the BEQ.

#### Empathy Quotient

The high alexithymia group scored (significantly) lower on the empathy scale (F(1,32) = 7.31, p = 0.01) but did not differ on the control items (F(1,32) = 0.86, p = 0.36).

#### Positive and Negative Affect Schedule

Groups did not differ on the positive (F(1,32) = 0.17, p = 0.68) and negative affect schedule (F(1,32) = 2.60, p = 0.12).

### Tasks

#### Micro Expression Training Tool

Alexithymic participants scored significantly lower on recognizing brief emotional expressions (see Table 2) (F(1,31) = 9.60, p = 0.004).

**Table 2:** Mean scores (%) (S.D.) on the cognitive-emotional tasks for the high and low alexithymic groups.

	Alexithymic	Non-Alexithymic	F	χ^2^	P
**Emotional learning**	79.7 (18.8)	84.9 (13.0)	0.90		0.35
**Identity learning**	88.7 (9.5)	92.1 (10.4)	0.99		0.33
**METT**	74.2 (16.6)	88.8 (9.8)	9.60		0.004*
**Affective Prosody Task**					
Prosody Accuracy	73.2 (13.9)	77.1 (12.5)	1.77		0.16
Prosody Reaction Time (ms)	4040.4 (640.7)	3875.9 (728.3)	0.59		0.68
Semantic Accuracy	87.5 (7.6)	88.4 (8.4)	0.32		0.86
Semantic Reaction Time (ms)	4519.1 (329.2)	4491.8 (522,5)	2.44		0.07
**Conflicting Beliefs and emotions**					
1^st^ order cognition	93.8 (9.1)	98.6 (3.4)		2.72	0.10
2^st^ order cognition	95.3 (7.7)	98.6 (4.0)		2.17	1.14
1^st^ order emotion	85.2 (9.9)	95.1 (8.7)		9.46	0.002*
2^nd^ order emotion	75.4 (15.4)	78.5 (24.2)		1.12	0.29
Control questions	97.3 (4.4)	98.1 (2.7)		0.25	0.87

* significant at p<0.005 level (2-tailed).

#### Associative Learning Task

The groups did not differ on their performance on the associative learning task. This was the case for both emotional learning (F(1,32) = 0.90, p = 0.35), and identity learning (F(1,32) = 0.99, p = 0.33) (see Table 2).

#### Affective Prosody Task

The affective prosody task revealed no differences between groups, not on accuracy in the prosody task (F(4,29) = 1.77, p = 0.16) and not on accuracy in the semantics task (F(4,29 = 0.32, p = 0.86). Groups also showed no differences in reaction times, neither in the prosody task (F(4,25) = 0.59, p = 0.68) nor in the semantics task, although there was a trend for alexithymic individuals to react slower in the latter (see Table 2) (F(4,29) = 2.44, p = 0.07).

#### Conflicting Beliefs and Emotions task

The high alexithymic group performed significantly worse on the first order emotion question of the conflicting beliefs and emotions vignettes (χ^2^ = 9.46, p = 0.002). There was no difference in performance on the first (χ^2^ = 2.72, p = 0.10) and second order cognition (χ^2^ = 2.17, p = 1.14) nor on second order emotion (χ^2^ = 1.12, p = 0.29). In addition, no group difference existed on the control questions (χ^2^ = 0.25, p = 0.87) (see Table 2). The inter rater reliability was high (r = 0.77).

## Discussion

In this study, we observed that healthy participants with relatively high levels of alexithymia used less efficient emotion regulation strategies (i.e. more suppression, less reappraisal). They also reported less expressivity and lower impulse strength. With regard to cognitive-emotional processing, the alexithymic individuals were impaired at rapid recognition of emotional information from faces and, on a higher level of processing, at emotional mentalizing. Surprisingly, there were no specific deficits in processing of emotional language. None of these group differences was attributable to mood differences (on which groups did not differ). Participants scored high on the cognitive factor of alexithymia and relatively normal on the emotionalizing factor.

With regard to the self report questionnaires, previous studies on emotion regulation in alexithymia have thus far focused on suppressive and repressive [8], [38] strategies. Suppression, as measured by the Illness Behaviour Questionnaire [39], correlates positively with two subscales of the TAS-20: difficulty expressing feelings to others and externally oriented thinking [40]. To our knowledge, our study is the first to investigate reappraisal as an additional emotion regulation strategy to suppression, as outlined in the influential model by Gross and John [20]. Alexithymic individuals had lower reappraisal scores and higher suppression scores on the emotion regulation questionnaire. This pattern has been associated with lower levels of well-being [20]. Because reappraisal occurs early in the emotion regulation process, before the emotion response tendencies have been fully generated, it can determine the entire upcoming emotional trajectory [20]. Our novel finding of the relationship between alexithymia and difficulties with reappraisal, suggests that an enhanced focus on reappraisal might be beneficial in the therapy of alexithymia.

The Berkeley Expressivity Questionnaire can differentiate between negative and positive emotion-expressive behavior and has been shown to have substantial correspondence with peer ratings [28]. The alexithymic group reported less behavioral expressivity for both positive and negative emotions. Additionally, their general strength of emotion response tendencies was weaker. These findings are in agreement with increased employment of suppressive emotion regulation strategies by the alexithymic group.

Furthermore, the alexithymic group reported lower levels of empathy. This corroborates and extends previous research investigating empathy and alexithymia: Guttman and Laporte [41] reported lower empathy, measured with the interpersonal reactivity index – IRI [42], in alexithymic participants, defined by the TAS-20. Alexithymic individuals scored lower on the subscales perspective taking, empathic concern and scored higher on personal distress. The same pattern was demonstrated in alexithymic students [17]. Our study demonstrated the same inverse relationship in healthy alexithymic individuals, but using other measures of alexithymia and empathy. The basis for this correlation may be that feeling empathy for another person requires understanding of the other's feeling which may in turn rely on knowing one's own feeling. This concept would imply that alexithymia undermines empathy (but a lack of empathy should not necessarily lead to alexithymia).

The behavioral emotional processing tasks extended the questionnaire findings from self to others. Questionnaires showed maladaptive processing of own emotions, while behavioral tasks showed that processing and recognizing of others' emotions was also, quite specifically, impaired. This was evident from both the METT and the *conflicting beliefs and emotion* task.

On the higher order emotion-processing task, the *conflicting beliefs and emotions task*, participants in the high alexithymic group displayed poor understanding of the first order emotional state. This can be explained by the ‘blindfeel’ hypothesis: alexithymia is characterized by a deficit in interoceptive awareness despite the fact that behavioral and autonomic reactivity are present. Alexithymics either feel nothing or do not recognize the feeling [43]. Similar to the case for empathy, knowing another person's feeling probably requires awareness of one's own feelings.

Both groups performed the same on the first order question about the conflicting belief. The same applied to the second order conflicting belief and the associated emotional state, but these latter questions were very difficult to interpret which may explain the lack of a difference. Both groups scored equally well on the control questions, which confirms that there was no difference in understanding of the story. Consistent with findings of Wastell and Taylor [44] who reported normal performance of alexithymic individuals in a false belief task, our findings confirm that the high alexithymic group was quite able to think about others in a different situation. Our results extend this by showing that the impairment is specific for emotions.

Interestingly, in a study employing the same task, patients with early amygdala damage also made more errors in emotional attributions [36]. As both patients with amygdala damage and high alexithymic individuals fail to attribute the correct emotional state to another person, it would be interesting to specifically investigate the role of the amygdala in alexithymia. Subclinical amygdala damage could be the neural basis for certain forms of alexithymia.

To some extent this has already been investigated but without confirming altered amygdala activation in alexithymia [17], [45], [46]. However, these studies did not specifically focus on the amygdala or used relatively small subject groups [45], [46] or did not specifically look at emotion processing [17]. It therefore remains an interesting possibility that subclinical amygdala damage could be the neural basis for certain forms of alexithymia.

Alexithymic individuals were not only impaired on higher order emotion processing but also on low level early processing of emotions. Lane et al. [8], already demonstrated that highly alexithymic participants have deficits in recognition of emotions. We observed that, after a training session, alexithymic individuals recognized micro-expressions less accurately than non-alexithymic participants. Thus, alexithymic individuals do not benefit from training in visual emotional features to such an extent that they can equal the performance of non-alexithymics'.

To extend studies on visual emotional processing, we included an auditory emotional task: judging the emotional tone of spoken language i.e. prosody. This emotional prosody task previously indicated deficits in Klinefelter [47] and schizophrenia [48] patients. The current study is, as far as we know, the first to examine affective prosody in alexithymia. Sentences had an emotional semantic content and were spoken with an incongruent prosody. Alexithymic individuals did not demonstrate problems with recognizing emotional content nor prosody. It thus seems that alexithymia is not specifically an emotional language-related problem. Lane et al. [43] suggested that in alexithymia emotional experience is blunted or absent in contrast to aprosodic individuals who experience emotions fully. Thus, these two disorders rely on different mechanisms.

On a more cognitive-emotional level high alexithymics were not impaired on associative emotional learning nor on identity learning. This is in disagreement with the notion that they have difficulties coupling words to emotional states [19]. It thus appears that the task does not correlate well with the verbalizing scale of the BVAQ. Possibly, this is the case because verbalizing requires associating words with one's own emotions whereas for this task words had to be associated with external images of facial expressions. Regarding any negative findings, it should be noted that neurophysiological differences may nevertheless be present. Specifically, Vermeulen et al. [49] found no effects of alexithymia on perception of emotional expressions, despite delayed neurophysiologic responses. Furthermore, the generalizability of this study is limited by the fact that only healthy university students participated, who on average function at a high level. Studies on people with very high levels of alexithymia, e.g. patients with psychosomatic complaints, may present different results.

A clinical implication of our findings might be that training or therapy focused on awareness, recognition and regulation of emotions might be beneficial for individuals with alexithymia. Greenberg [50] proposed one such approach, named “emotion-focused therapy”. In this approach, patients are taught how to become aware of their emotions, to understand their bodily reactions, and to express emotion in a context appropriate way. Although not specifically designed for alexithymic individuals, it could be tailored to each individual [50].

In sum, questionnaires indicated that participants scoring high on the cognitive component of alexithymia are characterized by suppressive rather than reappraisal strategies. In addition, alexithymic individuals showed specific deficits in emotional processing. Alexithymic individuals were impaired on recognition of briefly presented emotional expressions and on emotional mentalizing. No deficits were seen in processing of emotional language nor in associating words to emotional faces. This implies that the interaction of language and emotions might not be at the core of alexithymia. Future studies are necessary to explore the specific emotion processing difficulties in alexithymia. The use of brain imaging may help unravel brain mechanisms underlying emotional processing deficits in alexithymia. For example, Aleman [19] suggested that compromised interactive processing in hippocampal–amygdala circuits during emotional relational memory could underlie the verbalizing problems in alexithymia. Neuroimaging techniques and appropriate data analysis methods may enable us to shed more light on brain structures that are implicated in alexithymia.
